# CYP2C9 Genotype vs. Metabolic Phenotype for Individual Drug Dosing—A Correlation Analysis Using Flurbiprofen as Probe Drug

**DOI:** 10.1371/journal.pone.0120403

**Published:** 2015-03-16

**Authors:** Silvia Vogl, Roman W. Lutz, Gilbert Schönfelder, Werner K. Lutz

**Affiliations:** 1 Institute of Pharmacology and Toxicology, University of Würzburg, Würzburg, Germany; 2 Department of Clinical Pharmacology and Toxicology, Charité-Universitätsmedizin Berlin, Berlin, Germany; 3 Seminar for Statistics, Swiss Federal Institute of Technology, Zürich, Switzerland; 4 Federal Institute for Risk Assessment (BfR), Berlin, Germany; Tel Aviv University, Israel, ISRAEL

## Abstract

Currently, genotyping of patients for polymorphic enzymes responsible for metabolic elimination is considered a possibility to adjust drug dose levels. For a patient to profit from this procedure, the interindividual differences in drug metabolism within one genotype should be smaller than those between different genotypes. We studied a large cohort of healthy young adults (283 subjects), correlating their CYP2C9 genotype to a simple phenotyping metric, using flurbiprofen as probe drug. Genotyping was conducted for CYP2C9*1, *2, *3. The urinary metabolic ratio MR (concentration of CYP2C9-dependent metabolite divided by concentration of flurbiprofen) determined two hours after flurbiprofen (8.75 mg) administration served as phenotyping metric. Linear statistical models correlating genotype and phenotype provided highly significant allele-specific MR estimates of 0.596 for the wild type allele CYP2C9*1, 0.405 for CYP2C9*2 (68 % of wild type), and 0.113 for CYP2C9*3 (19 % of wild type). If these estimates were used for flurbiprofen dose adjustment, taking 100 % for genotype *1/*1, an average reduction to 84 %, 60 %, 68 %, 43 %, and 19 % would result for genotype *1/*2, *1/*3, *2/*2, *2/*3, and *3/*3, respectively. Due to the large individual variation within genotypes with coefficients of variation ≥ 20 % and supposing the normal distribution, one in three individuals would be out of the average optimum dose by more than 20 %, one in 20 would be 40 % off. Whether this problem also applies to other CYPs and other drugs has to be investigated case by case. Our data for the given example, however, puts the benefit of individual drug dosing to question, if it is exclusively based on genotype.

## Introduction

Individual differences in the rate of metabolic elimination of xenobiotics can be investigated by genotyping or phenotyping. In the attempt to minimize adverse drug reactions (ADR), the polymorphic CYP2C9 monooxygenase is one of the enzymes of interest. This cytochrome-P450 isoform takes part in the metabolism of approximately 15% of the clinically used drugs [1], and metabolizes predominantly small lipophilic/weakly acidic molecules. Some non-steroidal anti-inflammatory drugs (NSAIDs), oral anticoagulants, angiotensin II blockers and sulfonylurea hypoglycemic drugs share these features [2], and many authors recommend respective dose adjustments according to the CYP2C9 genotype of the patient [3–5]. For specific drugs like celecoxib or warfarin, genotype-dependent dose adjustments are already suggested in US FDA-approved drug labels [6].

In persons of Western European descent, three alleles have been identified, the wild type allele CYP2C9*1 with normal enzyme activity and a high allelic frequency of nearly 80%, and CYP2C9*2 and *3 with allele frequencies of approximately 13% and 7%, respectively, and reduced enzyme activity. As a consequence, approximately one third of all persons of Western European descent exhibit reduced CYP2C9 enzyme activity [4].

**Fig 1:**
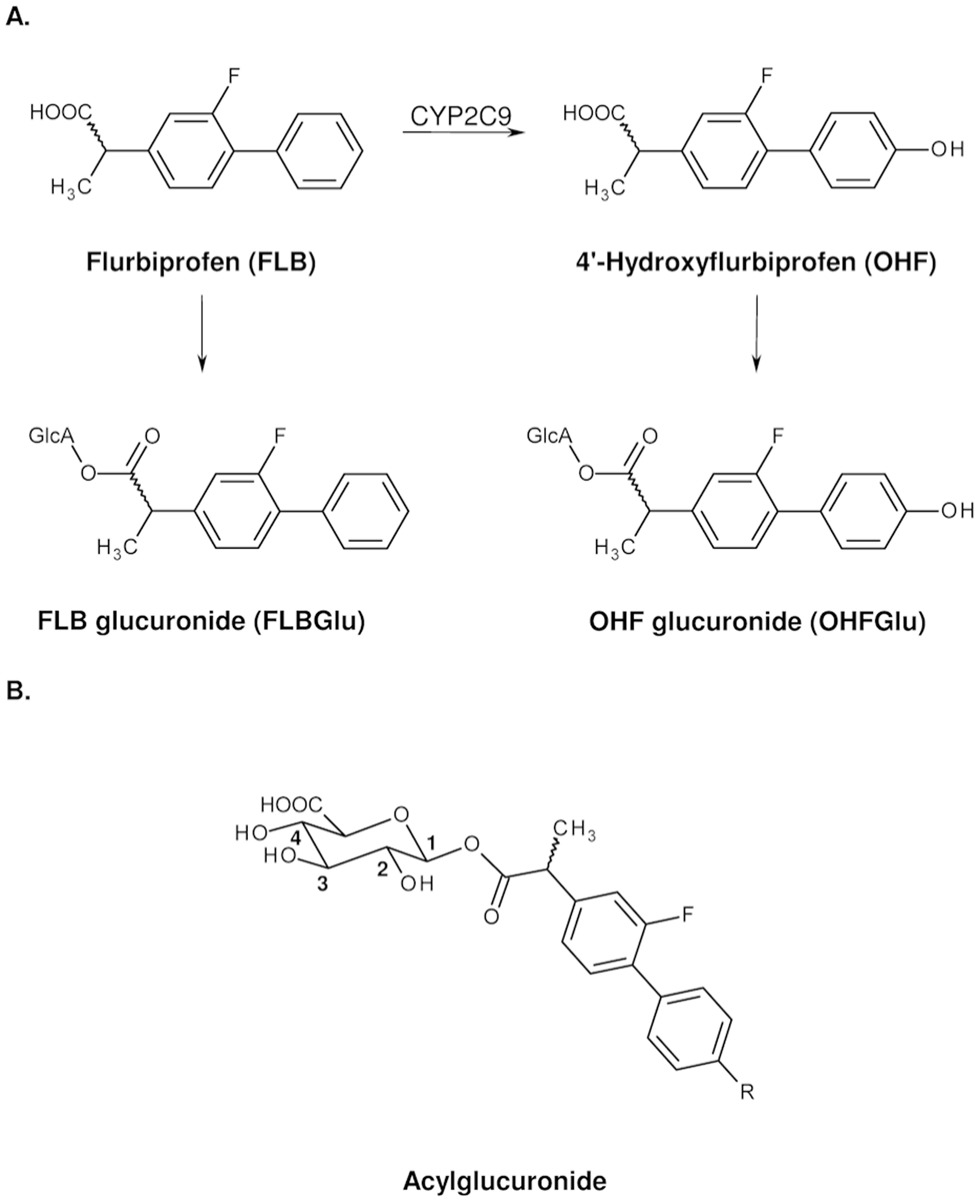
Flurbiprofen metabolism. A. Simplified metabolism of flurbiprofen in humans. B. FLB (R = H) and OHF (R = OH) acylglucuronides with positions of the glucuronic acid moiety.

Genotyping allows identification of the CYP2C9 allele status and is independent of environmental influences. This feature could likewise be considered a drawback, as this approach provides no information on the actual level of enzyme activity that may depend on numerous modulating influences. Phenotyping on the other hand, i.e., the direct analysis of metabolite ratios using appropriate probes, would take into account such factors. The NSAID flurbiprofen (FLB) is one of the possible probe drugs for CYP2C9 activity, based on the rate of hydroxylation of FLB to 4’-hydroxyflurbiprofen (OHF). Both FLB and OHF are conjugated with glucuronic acid to form acyl glucuronides (Fig. 1A). These acyl glucuronides can rearrange to structural isomers via acyl migration (Fig. 1B) [7].

FLB and all metabolites are excreted in urine. After glucuronide cleavage, it is therefore possible to measure the fraction of FLB that had been hydroxylated at the 4’-position by CYP2C9. The ratio of the urinary concentrations of hydroxylated metabolite and parent compound (metabolic ratio, MR), had been reported to be a suitable phenotyping index for CYP2C9 activity, using urine collected 0–8 h after ingestion of 50 mg FLB [8]. Other published phenotyping methods with FLB use even higher doses and measure the area under the plasma concentration time curves or MR in urine sampled for periods of up to 0–24 h after FLB ingestion [9–12]. Recently, it could be demonstrated that the MR measured in 5 μl blood (dried blood spot, DBS) drawn 2 h after administration of 50 mg FLB can also be used to assess CYP2C9 enzyme activity [13].

The correlation between genotype and phenotype is a matter of debate. For the coumarin anticoagulant warfarin, many studies compared genotypes of several genes and dose requirements, which led to more than 30 dosing algorithms based on CYP2C9 genotype besides Vitamin K epoxide reductase complex subunit 1 genotypes and other factors like e.g. height and age [14,15]. Quantitative estimations of CYP2C9 enzyme activities coded by the variant CYP2C9*2 and *3 alleles and subsequent genotype-based dose recommendations have already been published. However, they are mostly based on either *in-vitro* results, clinical studies with small healthy cohorts and/or heterogeneous cohorts of patients without consideration of concomitant medication, or meta analyses of these studies [4,16,17]. Only few data are available for cohorts where both typing procedures have been used on the same healthy persons, and there is a lack of statistical analysis of phenotypes to determine confidence limits for any estimated parameters [18]. Here, we provide this type of information for CYP2C9 activity by combining genotyping with phenotyping for a cohort of 283 healthy students, using FLB as probe drug.

## Results

### Allele frequencies and genotypes

The observed allele frequencies are 80.0% for CYP2C9*1, 11.8% for CYP2C9*2 and 8.1% for CYP2C9*3. The observed genotype frequencies are shown on the left-hand side of Table 1 and are consistent with those found in the literature [19]. About two thirds of our group were homozygous for the wild type allele *1, only one single individual was found to be a homozygous CYP2C9*3/*3 carrier.

**Table 1:** CYP2C9 genotypes and respective measured and estimated metabolic ratios for flurbiprofen.

		MR Measured		MR Estimated	
Genotype	Number/frequency	MR (mean ± SD)	Percent of wild type activity	MR (estimate ± SE)	Percent of wild type activity
CYP2C9*1/*1	181/64.0%	1.189 ± 0.314	= 100	1.192 ± 0.021	= 100
CYP2C9*1/*2	52/18.4%	1.005 ± 0.202	85	1.001 ± 0.033	84
CYP2C9*1/*3	39/13.8%	0.728 ± 0.256	61	0.709 ± 0.041	60
CYP2C9*2/*2	5/1.8%	0.834 ± 0.284	70	0.810 ± 0.066	68
CYP2C9*2/*3	5/1.8%	0.424 ± 0.095	36	0.518 ± 0.052	43
CYP2C9*3/*3	1/0.4%	0.096 single value	8	0.226 ± 0.084	19

Estimated allelic contributions: 0.596±0.010, 0.405±0.033, and 0.113±0.042 for CYP2C9*1, *2 and *3, respectively.

SD, standard deviation; SE, standard error; MR, metabolic ratio.

### Measured phenotypes (metabolic ratios)

**Fig 2:**
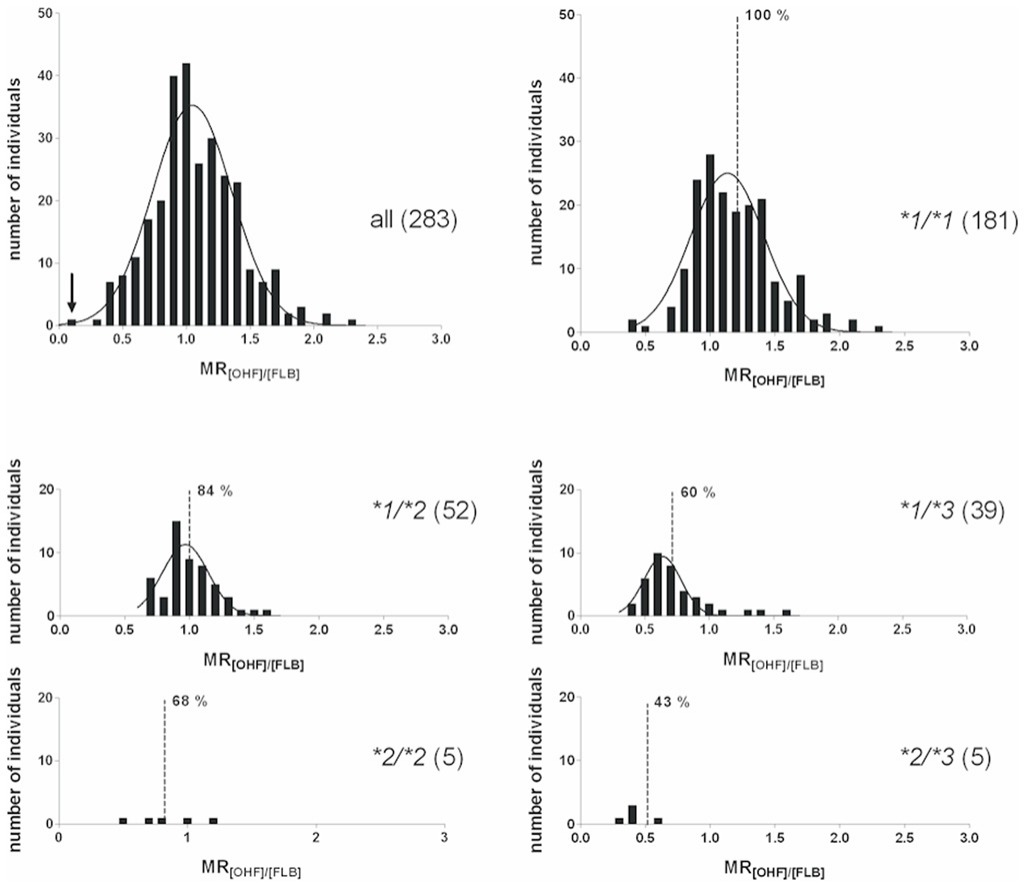
Histograms. Distribution of the metabolic ratio (MR; concentration of 4’-hydroxyflurbiprofen [OHF] divided by the concentration of flurbiprofen [FLB]) measured in urine 2 h after administration of flurbiprofen to 283 individuals. Histograms are shown for the entire study group and for the genotypes with more than one individual. One single individual with genotype CYP2C9*3/*3 showed an MR of 0.096 (highlighted by an arrow in the histogram). The dashed lines indicate the best estimates of model-derived MRs, together with the FLB dose adjustment in percent of wild type *1/*1 (= 100%).

Individual urinary FLB concentrations varied 79-fold, from 0.82 to 64.7 μM (median = 5.93 μM), OHF concentrations varied 209-fold from 0.39 to 81.1 μM (median = 6.10 μM). The distribution of the MRs (concentration of OHF divided by the concentration of FLB) is shown in Fig. 2 for all subjects (top left panel) and for five genotypes. Individual values spanned 24-fold from 0.096 to 2.32, with a mean of 1.07. The subject with the lowest metabolic activity was identified as a homozygous carrier of CYP2C9*3 (highlighted by an arrow in the histogram for all individuals, Fig. 2). The distributions appeared close to normal, some right skew was noted, most pronounced for the *1/*3 genotype. Mean and standard deviation of the measured MR values of six genotypes are shown in the center columns of Table 1. The means of the MRs of the five genotypes CYP2C9*1/*1, *1/*2, *1/*3, *2/*2, and *2/*3 differ statistically significantly (p < 0.001, Welch ANOVA, homogeneity of variances not assumed). Post-Hoc Tamhane’s T2 tests revealed statistically significant differences of means for every pair of genotypes, except for the tests against *2/*2.

### Estimation of allele-specific contributions to MR and of metabolic activity of genotypes

Linear models were applied to estimate the contributions of the three alleles to the metabolic activity of the individuals. Comparison of different data transformations indicated MR values as best choice. Best estimates of the allele-specific contributions to the measured MRs were 0.596 ± 0.010, 0.405 ± 0.033, and 0.113 ± 0.042, for CYP2C9*1, CYP2C9*2, and CYP2C9*3, respectively (best estimate ± standard error). The estimates were statistically significant with p < 10^-15^ for CYP2C9*1 and CYP2C9*2, and p = 0.007 for CYP2C9*3.

As the MR is proportional to the metabolic activity, the estimated allelic coefficients for CYP2C9*2 and CYP2C9*3 can be expressed in percent of the metabolic activity of the wild type allele CYP2C9*1 taken as 100%. Therefore, CYP2C9*2 allele accounts for 68% activity of wild type, CYP2C9*3 can be assigned an activity of 19%.

Neither by adding a term “homozygous genotype”, nor using a model based on six genotypes instead of the sum of alleles, could the regression be improved. This result is consistent with the assumption that the allelic contributions to MR are independent of each other, and indicates a co-dominant expression.

The metabolic activity of the six genotypes equals the sum of the model-derived allele-specific contributions and is shown in the right-hand side of Table 1. Expressed as percent of the wild type genotype CYP2C9*1/*1, the metabolic activity is 84%, 60%, 68%, 43%, and 19% for *1/*2, *1/*3, *2/*2, *2/*3, and *3/*3, respectively. The model-derived relative standard errors are between 1.7% and 10.1% of the estimates for all genotypes except *3/*3 (37%), which corroborates the significance of the model.

### Variability of measured MR within a genotype

The narrow confidence interval for the prediction of a genotype-specific MR contrasts with the large interindividual variation of measured MRs within a given genotype. The coefficients of variation are 26%, 20%, 35%, 34%, and 22%, for genotypes *1/*1, *1/*2, *1/*3, *2/*2, and *2/*3, respectively. This indicates that the individual rate of hydroxylation of FLB can hardly be predicted on the basis of the *CYP2C9* genotype alone, even though hydroxylation of flurbiprofen is considered to be solely catalyzed by this enzyme [20,21].

In the search of factors that modulate the individual metabolic ratio we analyzed the questionnaires completed by the study group. Testing for any effect of oral contraception, smoking (induction?) or vegetarian diet did not reveal any significant difference. There was no difference either between males and females. A particular look at outliers within a genotype did not either indicate important confounding factors. One *1/*3 individual was more than three standard deviations above the average of this genotype and showed a measured MR of 1.611. The questionnaire filled in by this 28-year old female indicated oral contraception (ethinylestradiol/dienogest), but no conditions such as other drugs or any other factor listed above.

## Discussion

Several studies postulated the influence of the CYP2C9 genotype on maintenance doses and risk for specific adverse drug reactions (ADRs) of CYP2C9 substrates. It could for instance be shown that carriers of the variant *CYP2C9* alleles *2 and *3 need significantly lower doses of the coumarin anticoagulant warfarin and are, as a group, more prone to respective ADRs, such as bleeding complications [22–25]. However, these studies are based on responses in groups of warfarin-treated patients. The present study, on the other hand, was designed to investigate on an individual level the predictive power of the CYP2C9 genotype for the metabolic elimination of a probe drug in a healthy group of young volunteers.

### Development of phenotyping method

Dose reduction from 50 mg to 8.75 mg FLB was possible thanks to the development of a new LC-MS/MS analytical method. To our knowledge our method is the most sensitive ever published for FLB and OHF in urine. The relative phenotyping outcome should be independent from the dosage, as long as the dose does not exceed the linear range of the CYP2C9 enzyme dose-response curve. Therefore the FLB dose reduction should only have beneficial effects for the safety of the phenotyping method without influencing the relative phenotyping outcome. It was shown by Zgheib et al. that the correlation between the urinary metabolic ratio of FLB and the OHF formation clearance calculated as the total amount of OHF recovered in urine (24 h) divided by the FLB AUC_0–24_ is comparable for each urine collection interval they evaluated (0–2, 0–4, 0–6, 0–8, 0–10, 0–12 and 0–24 h after drug administration) [8]. Based on this work we shortened the waiting period from 8 to 2 hours as it resulted in a more practicable working schedule.

### Estimation of allele-specific contribution to the MR

Our linear model to estimate the contributions of the three alleles to the MR of the six genotypes provided highly significant results. Statistically significant differences of means could be detected across the five genotypes with more than one subject. The metabolic activity attributed to the alleles *2 and *3, expressed relative to 100% for the wild type allele*1, was 68%, and 19%, respectively. A variety of values have been given in the literature. For CYP2C9*2, a reduction of the rate of metabolism to approximately 50% of the wild type had been proposed [17]. Another publication reported on a reduction by ~20–30% [26]. For CYP2C9*3, a loss of up to 70% of the enzyme activity had been published [26]. Most literature values had been determined *in-vitro*. This may explain some of the differences to our values. A recent calculation based on meta-analyses of *in-vivo* studies estimates the fraction of activity for the different CYP2C9 genotypes to be 100% (*1/*1), 82% (*1/*2), 56% (*1/*3), 70% (*2/*2), 36% (*2/*3) and 13% (*3/*3) [16]. Our results (Table 1) are in good agreement with these values. This is in line with the suggested 80% FLB dose reduction recommendation for CYP2C9*3/*3 carriers [10,16], as well as with the respective recommendation for another CYP2C9 substrate, the hypoglycemic drug tolbutamide [18].

### Interindividual variation vs. genotype-specific dose adjustment

One of the most critical findings in our study is the discrepancy between the high significance and small relative errors of the MR predictions and the wide variability of individual MR measures. Even though our study was conducted in a cohort of healthy volunteers with a very small age range and no concomitant medication known or suspected to influence FLB pharmacokinetics, the MRs in each genotype subgroup varied widely.

In the histograms of Fig. 2 we indicated the model-estimated MRs for the different genotypes by vertical lines. Based on the assumption that suggested dose levels are proportional to the MR, the genotype-specific recommendations for the maintenance dose in percent of wild type (CYP2C9*1/*1 = 100%), are also given. A look at the histograms around these values reveals the problem of using the same dose recommendation for all carriers of a given genotype.

In order to express this problem in quantitative terms we translated the standard deviations of measured MR into the dose scale. Assuming normal distribution and considering that ± 1 standard deviation comprise about two thirds of the values, it follows that one of three individuals is expected to have its optimum dose outside a dose range of 78–122% for *1/*1, 67–101% for *1/*2, 30–90% for *1/*3, 39–97% for *2/*2, and 24–62% for *2/*3. It is obvious that dose adjustment on the basis of the genotype alone would not be justified.

### MR-modulating factors beyond the CYP2C9 genotype

Part of the large individual variation of the phenotyping metric observed in our study might be explained by our study design, i.e. the choice of probe drug and the relatively short urine collection time of 2 h. Tolbutamide, another probe drug for CYP2C9 is thought to be more sensitive than FLB [9]. However, tolbutamide as a probe drug holds two problems. Firstly, tolbutamide administration is associated with the risk of hypoglycaemia and secondly only a very small amount of unchanged tolbutamide is excreted in urine which limits reliable measurement [8,9]. The use of very small tolbutamide doses can reduce the risk of hypoglycaemia, but then the small amount of unchanged drug in urine can be problematic. To minimize the risk of adverse drug reactions, we used the best and safest alternative probe drug which is in our opinion flurbiprofen. Flurbiprofen was shown to be exclusively metabolized to OHF by CYP2C9, and proposed as a valid *in-vitro* probe drug for CYP2C9 [21,27,28]. It was thereafter also used and validated as a CYP2C9 *in-vivo* probe drug [8,10,13,29], including a study which showed that two 200 mg doses of the potent CYP2C9 inhibitor fluconazole caused a reduction of FLB clearance to approximately 55% of placebo values [11]. Additionally, there are no genetically polymorphic transporters known to influence FLB uptake or excretion. As mentioned before, the 0–2 h urine collection interval has been demonstrated to be as valid as the 0–8 h and the 0–24 h interval used in other FLB phenotyping procedures [8]. Therefore it is unlikely that our study design is the main reason for the large individual variation. Other factors have to be considered as well, most importantly those influencing the available concentration of active CYP2C9 enzyme. The limited information about known CYP inducers/inhibitors from the questionnaire (smoking; consumption of grapefruit juice) did not give a lead. The amount of active CYP2C9 in general depends on the level of gene expression, the rates of mRNA transcription and degradation, the degradation of the protein and the presence of enzyme inhibitors/antagonists. Post-transcriptional regulation of CYP2C isoforms by microRNAs has already been demonstrated [30]. The amount of protein of other CYP isoforms (CYP2D6, CYP3A4) might also influence CYP2C9 activity via protein-protein interactions. This is at least suggested by i*n-vitro* findings in human hepatocytes and reconstituted systems [31–33]. Recently, Temesvari et al. showed for carriers of the CYP2C9*1/*1 genotype that the CYP2C9 mRNA content of leukocytes is directly correlated to the ability of liver tissue to hydroxylate the established CYP2C9 probe drug tolbutamide. They demonstrated that CYP2C9 protein levels and tolbutamide hydroxylation capacity varies highly even between subjects of the same genotype [34].

Our study examines the contribution of CYP2C9 alleles to the hydroxylation of FLB and cannot be transferred uncritically to other enzymes or drugs. Nevertheless, our data indicate that phenotyping provides a more direct link to the individual performance in metabolic elimination, which cautions against premature dose recommendations solely based on genotyping enzymes involved in metabolism. An inclusion of measures of CYP2C9 protein amount in the liver—e.g. by measuring the CYP2C9 mRNA content of leukocytes—in addition to CYP2C9 genotyping might be advantageous to predict the phenotype of an individual. It would also be very useful to correlate two CYP2C9 phenotyping methods without intermediary genotyping to examine the influence of the used probe drug on the enzyme activity measurement, thereby further establishing the transferability of these measurements to other CYP2C9 substrates. This could ultimately lead to more nuanced dose recommendations based on standardized phenotyping methods instead of genotyping.

## Methods

### Probe drug, chemicals and enzymes

Dobendan Direkt lozenges (Boots Healthcare, Hamburg, Germany) were provided by Klosterfrau Healthcare Group, Köln, Germany. Water HPLC-grade, acetonitrile HPLC-grade, and methanol HPLC-grade were from Roth, Karlsruhe, Germany. Human CYP2C9*1 Supersomes were from BD Biosciences, Woburn, USA. Flurbiprofen (FLB) was purchased from Sigma-Aldrich, Steinheim, Germany, flurbiprofen-d_3_ (FLB-d_3_) was from TRC, Toronto, Canada. 4-Hydroxyflurbiprofen was a gift from the Dept. of Experimental and Clinical Pharmacology, College of Pharmacy, University of Minnesota, Minneapolis, USA. Primers were purchased from Invitrogen GmbH, Darmstadt, Germany. LongAmp Taq polymerase and restriction endonucleases AvaII, NsiI, and KpnI together with the respective ready-to-use buffers were purchased from New England Biolabs, Frankfurt a. Main, Germany.

### Subjects and treatment

In a laboratory course in pharmacology and toxicology altogether 291 students of European descent of the Würzburg University registered for phenotyping and genotyping for a study that had been approved by the Medical Faculty’s Ethical Committee Würzburg University. Of those, 8 subjects could not be successfully genotyped. The resulting cohort of 283 subjects consisted of 201 females with a mean ± SD age of 23 ± 2 years (range, 20–34) and 82 males aged 24 ± 2 years (range, 21–33). All tested subjects were of good health, did not ingest drugs with known influence on flurbiprofen metabolism, and gave informed written consent. They also answered a questionnaire covering the following topics: smoking behaviour, administered drugs, consumption of grapefruit juice, diet (e.g. vegetarian), and diagnosed liver dysfunction.

After voiding the bladder, the participants ingested 8.75 mg FLB as a lozenge. After 2 h, urine samples were collected, divided into aliquots, and stored at -20°C. According to the study design, which also designated phenotyping for CYP2D6, 20 mg dextromethorphan in water (DEX, as dextromethorphan hydrobromide monohydrate, Fagron, Barsbüttel, Germany) had been administered simultaneously. Comprehensive interaction studies in initial studies had shown that there was no metabolic interaction between DEX and FLB at the dosage used. In addition to our own studies, it has been shown recently that FLB can be incorporated into a 4-Drug CYP phenotyping cocktail containing DEX without detectable influence of DEX on FLB metabolic ratio [35].

10 mL whole blood was taken from each participant using the winged infusion set Venofix (B.Braun, Melsungen, Germany) together with multi adapter and S-Monovette (Sarstedt, Nümbrecht, Germany), either shortly after probe drug ingestion or on another study day.

### Phenotyping

In order to use a lower FLB dose and a shorter time between ingestion and urine sampling than in the mentioned publications, we first improved the analytical procedure to (i) use urine without prior extraction of analytes, (ii) establish a new fast glucuronide cleavage method that leads to minimal dilution of the samples, and (iii) develop more sensitive LC-MS/MS analytics to simultaneously quantify the concentration of FLB and OHF. Using this methodology, we determined the MR 2 h after application of an 8.75 mg FLB lozenge (Dobendan Direkt).

**Standards**. FLB, OHF and flurbiprofen-d_3_ (FLB-d_3_) were dissolved in acetonitrile (1 mg/mL) and stored at -20°C. 4’-Hydroxyflurbiprofen-d_3_ (OHF-d_3_) was synthesized by incubation of FLB-d_3_ with Human CYP2C9*1 Supersomes and subsequently purified via HPLC.

Urine standard samples were prepared by spiking 200 μL blank urine with 5 μL of a standard solution. In that manner, 9 calibration standards were produced, containing FLB and OHF from 50 pmol/ml to 29 nmol/ml urine (50 nM, 0.1 μM, 0.2 μM, 1 μM, 2μM, 5 μM, 10 μM, 20 μM, 29 μM). The standard samples were treated like the actual samples as described in the following paragraph.

**Urine sample preparation**. FLB- and OHF-acylglucuronides can rearrange to structural isomers via acyl migration of the drug [7]. Glucuronide cleavage with ß-glucuronidase is only possible for 1ß-glucuronides. To allow for complete glucuronide cleavage with minimal sample dilution, a new cleaving method was developed based on the basic saponification of esters. Instead of extracting analytes after glucuronide cleavage, the treated urine was injected directly. The new method was verified in that neither FLB glucuronide nor OHF glucuronide were detectable by LC-MS/MS. By measuring respective concentrations of FLB and OHF before and after the treatment it was also determined that FLB and OHF were stable under the used conditions. Cleavage procedure: after centrifuging the urine samples for 10 min at 22 000 x g, 1 mL of the supernatant was mixed with 60 μl 3N NaOH, incubated at 90°C for 10 min, cooled on ice and neutralized with 30 μl 6N HCl. Afterwards, 5 μl of a mixture of internal standards (containing 100 pmol FLB-d_3_ and 25 pmol OHF-d_3_) were added to 75 μl of the treated sample and the mixture was analyzed directly. Samples with FLB and/or OHF concentrations exceeding 29 μM (highest concentration of standard curve) were diluted with water 1:10 and measured again.

**Liquid chromatography—mass spectrometry**. The analyses were performed using an Agilent 1100 LC system (Agilent, Böblingen, Germany) coupled to a QTRAP 2000 mass spectrometer of Applied Biosystems (Darmstadt, Germany) equipped with a Turbo Ionspray source set on the following parameters: IS 4000V, TEM 400°C, N_2_ as curtain gas (40), gas 1 (45), gas 2 (65) and collision gas (CAD = 4). Compound specific parameters were obtained by infusion of the standards using the quantitative optimization function of Analyst software 1.4.2 (Applied Biosystems).

For LC, a reprosil-pur phenyl 3μ column, 100 mm×2 mm with a corresponding guard cartridge was used (Dr. Maisch HPLC GmbH, Ammerbuch-Entringen, Germany). The mobile phase consisted of (A) 10 mM NH_4_OAc buffer pH 5 and (B) acetonitrile/methanol (1/1). The flow rate was 200 μL/min and the injection volume 10 μl. For the analysis of FLB and OHF, the following conditions were used: 80% A isocratic for 1 min, followed by a linear gradient to 10% A within 1 min then isocratic 10% A for 5 min. Within 1 min the ratio was set back to starting conditions and the system was equilibrated for 8 min. Analytes were recorded by multiple reaction monitoring in the negative ion mode. The compound specific parameters for FLB, OHF, FLB-d_3_ and OHF-d_3_ and their respective retention times are given in Table 2. Three replicate calibration curves were established. A metabolic ratio (MR) was calculated for all individuals by dividing the urinary concentration of the CYP2C9-dependent metabolite OHF by the concentration of the parent drug FLB: MR = [OHF]/[FLB]. This means that a high MR value corresponds to a high metabolic activity.

**Table 2:** Compound specific liquid chromatography/ mass spectrometry parameters for flurbiprofen (FLB), 4’-hydroxyflurbiprofen (OHF) and the respective deuterated internal standards FLB-d_3_ and OHF-d_3_.

Compound	Transition	DP (V)	CE (V)	Retention time (min)
FLB	243 → 199	-26	-10	8.1
OHF	259 → 215	-16	-12	7.7
FLB-d_3_	246 → 202	-21	-12	8.1
OHF-d_3_	262 → 218	-21	-12	7.7

DP, declustering potential; CE, collision energy.

**Analytical performance**. Calibration curves for FLB and OHF were linear from 50 nM to 29 μM with correlation coefficients of 0.992 and 0.994 and slopes of 0.0046 and 0.0025, respectively. Linear regression and 1/x weighing gave the best fit for accuracy with 86–120% for FLB and 85–120% for OHF. According to FDA guidelines for a limit of quantification LOQ (response at least 5 times the response of the blank and an identifiable, discrete, and reproducible response at LOQ with a precision of 20% and an accuracy of 80–120%), the LOQ was determined to be 50 nM for both FLB and OHF in urine. The method showed high intra- and interday precision and accuracy (coefficients of variation <10%, accuracy: 99–106%).

**Genotyping**. Genomic DNA from 10 ml peripheral blood was either isolated from buffy coat using the NucleoSpin Kit (Macherey-Nagel GmbH & Co. KG, Düren, Germany) or from fresh or frozen blood, employing a conventional salting out procedure. Genotyping for CYP2C9*2 (3608C>T, position referring to gene GenBank: AL359672.19) and CYP2C9*3 (42614A>C) was performed by polymerase chain reaction followed by restriction enzyme analysis, as validated by Sullivan-Klose et al [36]. Amplicons including gene position 3608 were digested with AvaII and those containing position 42614 with NsiI and KpnI. All alleles that were negative for the nucleotide substitutions at position 3608 (*2), and 42614 (*3) were presumed to be CYP2C9*1.

**Statistics**. The R-package was used for linear regression. It is available as Free Software under the terms of the Free Software Foundation’s GNU General Public License in source code form. Download is available at http://www.r-project.org. For correlation between genotype and phenotype, we used linear models to estimate the contributions of the alleles to the observed individual metabolic activity. MR values as well as different transformations [1/MR, log_10_(MR), -log_10_(MR)] were used for best correlation. The general form of the model was lm (MR ~ -1 + allel1 + allel2 + allel3). The term-1 stands for regression without intercept, which is required because the three explaining variables are not independent. Two models with interaction terms were also tested. One included a term for homozygous situations, the other distinguished between the six genotypes. Model-derived estimated MRs were calculated by summing up the estimated contributions of the alleles comprising the genotype. Histograms were generated with GraphPad Prism Software version 5 (GraphPad Software, Inc., CA, USA). All other statistical analyses were performed using SPSS Statistics Version 21.2 (IBM, Inc., NY, USA). All tests were two-tailed, and a probability of p < 0.05 was considered significant.

## Supporting Information

S1 Dataset Condensed dataset for 283 subjects. (XLSX)
