# Correction: CYP2C9 Genotype vs. Metabolic Phenotype for Individual Drug Dosing—A Correlation Analysis Using Flurbiprofen as Probe Drug

**DOI:** 10.1371/journal.pone.0126329

**Published:** 2015-04-13

**Authors:** Silvia Vogl, Roman W. Lutz, Gilbert Schönfelder, Werner K. Lutz

There is an error in the third sentence of the second paragraph of the “Subjects and treatment” subsection of the Methods. The correct sentence is: According to the study design, which also designated phenotyping for CYP2D6, 10 mg dextromethorphan in water (DEX, as dextromethorphan hydrobromide monohydrate, Fagron, Barsbüttel, Germany) had been administered simultaneously.
